# Formoterol as reliever medication in asthma: a *post-hoc* analysis of the subgroup of the RELIEF study in East Asia

**DOI:** 10.1186/s12890-015-0166-0

**Published:** 2016-01-12

**Authors:** Qi Jian Cheng, Shao-Guang Huang, Yu Zhi Chen, Jiang-Tao Lin, Xin Zhou, Bao-Yuan Chen, Yu-Lin Feng, Xia Ling, Malcolm R. Sears, on behalf of the RELIEF Asia Study investigators

**Affiliations:** Department of Pulmonary Disease, Ruijin Hospital, Shanghai Jiao Tong University, No.150 Wu Yi Road, Shanghai, 200025 China; Capital Institute of Pediatrics, Beijing, China; Department of Respiratory Diseases, China-Japan Friendship Hospital, Beijing, China; Department of Respiratory Medicine, Shanghai First People’s Hospital, Shanghai Jiao Tong University, Shanghai, China; Department of Respiratory Disease, Tianjin Medical University General Hospital, Tianjin, China; Department of Respiratory Medicine, West China Hospital, Sichuan University, Chengdu, Sichuan China; Medical and Regulatory Affairs, AstraZeneca China, Shanghai, 201203 China; Michael G DeGroote School of Medicine, Faculty of Health Sciences, McMaster University, Hamilton, ON Canada

**Keywords:** Asthma, Beta-agonist, Bronchodilator, Formoterol, Safety

## Abstract

**Background:**

As-needed formoterol can effectively relieve asthma symptoms. Since budesonide/formoterol is available as maintenance and reliever therapy in Asia, formoterol is now being used as-needed, but always with concomitant inhaled corticosteroids. The objective of this analysis was to assess the safety and efficacy of formoterol therapy in patients in East Asia (China, Indonesia, Korea, the Philippines and Singapore) with asthma.

**Methods:**

*Post-hoc* analyses of data from the East Asian population of the RELIEF (REal LIfe EFfectiveness of Oxis® Turbuhaler® as-needed in asthmatic patients; study identification code: SD-037-0699) study were performed.

**Results:**

This sub-group comprised 2834 randomised patients (formoterol *n* = 1418; salbutamol *n* = 1416) with mean age 35 years; 50.7 % were male. 2678 patients completed the study. There was no significant difference in the total number of adverse events (AEs) reported in the formoterol and salbutamol groups (21.3 % vs 20.9 % of patients; *p* = 0.813), nor in the total number of serious AEs and/or discontinuations due to AEs (4.6 % vs 5.5 %, respectively; *p* = 0.323). Compared with salbutamol, formoterol was associated with a significantly longer time to first exacerbation (hazard ratio 0.86; *p* = 0.023) and a 14 % reduction in the risk of any exacerbation (*p* < 0.05). Relative to salbutamol, mean adjusted reliever medication use throughout the study was significantly lower in the formoterol group (*p* = 0.017) and the risk of increased asthma medication use was 20 % lower with formoterol (*p* = 0.005).

**Conclusions:**

Among patients with asthma in East Asia, as-needed formoterol and salbutamol had similar safety profiles but, compared with salbutamol, formoterol reduced the risk of exacerbations, increased the time to first exacerbation and reduced the need for reliever medication.

**Electronic supplementary material:**

The online version of this article (doi:10.1186/s12890-015-0166-0) contains supplementary material, which is available to authorized users.

## Background

Current guidelines from the Global INitiative for Asthma (GINA) recommend an inhaled short-acting β_2_-agonist (SABA), such as salbutamol, terbutaline, fenoterol, levalbuterol, reproterol or pirbuterol, for the relief of symptomatic bronchoconstriction [1]. Formoterol is a long-acting β_2_-agonist (LABA) with a rapid onset of action and is recommended by GINA as an effective reliever medication in patients on regular controller therapy with inhaled corticosteroids (ICS) [1]. As-needed formoterol has previously been assessed [2, 3] and has been shown to effectively relieve asthma symptoms; a systematic review showed that formoterol used as reliever therapy reduced the number of exacerbations requiring oral corticosteroid treatment [4].

To determine the comparative efficacy and safety of formoterol and salbutamol as reliever therapy in patients with asthma, the global RELIEF study (REal LIfe EFfectiveness of Oxis® Turbuhaler® as-needed in asthmatic patients) was conducted [5]. In this 6-month, international, randomised, parallel-group study more than 18,000 adults and children with asthma from 24 countries used either formoterol or salbutamol as reliever therapy. The study was designed to mimic real-world clinical practice and therefore was open-label (i.e. no blinding), had minimal entry criteria and no run-in period; patient adherence to treatment was not monitored, as treatment was provided as-needed. The study included a large group of patients of varying age and asthma severity and who were receiving various maintenance medications. Overall, the safety profile of formoterol was similar to that of salbutamol (i.e. no significant differences in adverse events [AEs], serious AEs [SAEs] and discontinuations due to AEs [DAEs]), but patients treated with formoterol experienced a longer time to first exacerbation and required less as-needed and maintenance medication.

The availability of budesonide/formoterol as a maintenance and reliever therapy (Symbicort® SMART™) in countries in the Asia-Pacific region, including China, Japan, the Philippines and Thailand, means that formoterol in combination with ICS is now being used by these patients as a reliever therapy. Therefore, it is important to understand the safety of formoterol in such patients. Since limited real-world data exist regarding the comparative safety and efficacy of formoterol and salbutamol reliever medication in patients in East-Asia, a *post-hoc* analysis was performed to examine safety and efficacy outcomes among patients in the RELIEF study recruited from East-Asian countries.

## Methods

### Study design

The methodology and primary results from the RELIEF study (AstraZeneca study code: SD-037-0699) have been reported previously [5]. Briefly, an international, randomised, open-label, parallel-group, real-world study was performed in 24 countries to examine the safety and effectiveness of formoterol or salbutamol as reliever medication for asthma; >18,000 patients with varying degrees of asthma severity, background medication history and aged ≥6 years were randomised to receive formoterol or salbutamol as-needed for 6 months.

### Patient population

This *post-hoc* analysis includes data from the ‘eastern region’ subgroup, which comprised only patients recruited from China, Indonesia, Korea, the Philippines and Singapore. All findings described herein are derived from this population (unless otherwise stated) and written informed consent was obtained from, or on behalf of, all patients at the time the RELIEF study was conducted. Approval for this study was obtained from all relevant regulatory agencies. The study protocol was approved by the Independent Ethics Committee at the leading site (Ruijin Hospital, Ministry of Health), following approval by all local ethics committees at all study centres (Additional file 1: Table S1).

### Treatments

Patients were randomised to receive either formoterol (Oxis® Turbuhaler®; AstraZeneca, Lund, Sweden; 4.5 μg per delivered dose) or salbutamol (pressurised metered-dose inhaler [pMDI]/dry powder inhaler [DPI]; Diskhaler® [200 μg per dose; Ventolin™, GlaxoSmithKline, Uxbridge, UK], Diskus® [200 μg per dose; Ventolin™, GlaxoSmithKline, Uxbridge, UK] or Turbuhaler® [100 μg per dose; Inspiryl®, AstraZeneca, Lund, Sweden]), treatment was available to patients on the first day of the study immediately after randomization. Since these therapies were provided on an as-needed basis, compliance was not measured.

### Assessments

Clinic visits occurred at study entry and after 1, 3 and 6 months of treatment. The study was divided into three periods: Period 1 (study entry to end of first month), Period 2 (second and third months) and Period 3 (remainder of study).

The primary safety variables were asthma-related and non-asthma-related AEs, SAEs and DAEs. SAEs were defined as: any event causing hospitalization, prolongation of hospitalization, significant or persistent disability or congenital abnormality, any life-threatening condition or death. DAEs could be caused by serious or non-serious AEs.

The following events were defined as exacerbations due to deterioration of asthma: (1) any increase in maintenance asthma medication; (2) ≥5 days of oral corticosteroid treatment; (3) emergency treatment with corticosteroid injection or nebulised β_2_-agonist; (4) hospitalization. Severe exacerbations were defined as any one of events 2–4. Efficacy variables included time to first exacerbation; numbers of patients with exacerbations (all types); change in use of study medication; days with asthma symptoms.

### Statistical analyses

Statistical analyses were performed by intention-to-treat. A logistic regression model with factor treatment was applied within each stratum for the analysis of number of patients with at least one AE/SAE/DAE/asthma exacerbation. Results are expressed as odds ratios (ORs) with 95 % confidence intervals (CIs). The time to first asthma exacerbation was compared between treatments using a Cox proportional hazards model with factors treatment, stratum and interaction treatment by stratum. Results are expressed as hazard ratios (HRs) with 95 % CIs within each stratum, with the p-value for the interaction test. Time to first exacerbation (of any type) is presented in a Kaplan–Meier plot. Use of study medication and asthma symptom days were compared between treatments using a linear mixed-effects model; within this model adjustments were made for treatment, period and interaction treatment by period, for each stratum separately. The limited number of patients in this subgroup precluded analysis stratified by age or severity.

## Results

### Patients

The subgroup of patients recruited from East-Asian countries comprised 2834 patients (*n* = 1418 in the formoterol group and *n* = 1416 in the salbutamol group); 2678 (94.5 %) patients completed the study (1352 and 1326, respectively). Reasons for not completing the study included: not receiving treatment or no data on treatment (*n* = 16 and *n* = 23 in the formoterol and salbutamol treatment groups, respectively); discontinuation due to AEs (*n* = 15 and *n* = 17), lost to follow-up (*n* = 19 and *n* = 31), eligibility criteria not fulfilled (*n* = 0 and *n* = 1) or other reasons (not specified; *n* = 16 and *n* = 18).

The majority of patients were of Oriental descent (98.3 %). The mean age was 35 (range 5–85) years and 50.7 % were male. Patient baseline demographics/characteristics were evenly distributed between the two groups (Table 1). The demographics of this subgroup were similar to those of the entire study population [5] and to patients in other regional subgroups (Additional file 1: Table S2).

**Table 1:** Demographic and baseline clinical characteristics

Characteristic	Formoterol	Salbutamol	All
	(*n* = 1418)	(*n* = 1416)	(*n* = 2834)
Male	736 (51.9)	700 (49.4)	1436 (50.7)
Mean age, years (range)	35 (5–81)	36 (6–85)	35 (5–85)
Age groups			
Children ≤11 years	254 (17.9)	258 (18.2)	512 (18.1)
Adolescents 12–17 years	121 (8.5)	111 (7.8)	232 (8.2)
Adults 18–64 years	957 (67.5)	946 (66.8)	1903 (67.1)
Elderly ≥65 years	86 (6.1)	101 (7.1)	187 (6.6)
Race			
Oriental	1397 (98.5)	1389 (98.1)	2786 (98.3)
Caucasian	4 (0.3)	8 (0.6)	12 (0.4)
Other	17 (1.2)	19 (1.3)	36 (1.3)
Asthma severity (judged by asthma medication level)*			
Intermittent	206 (14.5)	201 (14.2)	407 (14.4)
Mild	740 (52.2)	713 (50.4)	1453 (51.3)
Moderate	299 (21.1)	318 (22.5)	617 (21.8)
Severe	173 (12.2)	184 (13.0)	357 (12.6)
Regular smoker	158 (11.1)	148 (10.5)	306 (10.8)

Data are presented as n (%) patients, unless otherwise stated

* Intermittent: no maintenance treatment; mild: inhaled corticosteroid (ICS) <500 μg/day (<400 μg/day in children) or a regular long-acting β_2_-agonist (LABA), cromone, theophylline or leukotriene modifier; moderate: ICS alone at any dose ≥500 μg/day (≥400 μg/day in children), or ICS 500–800 μg/day (400–800 μg/day in children) in combination with LABA, theophylline or leukotriene modifier; severe: ICS >800 μg/day in combination with LABA, theophylline, leukotriene modifier, or oral corticosteroids

Prior to randomization, a similar proportion of patients in each treatment group were receiving treatment for asthma. The majority of patients in each treatment group were prescribed ICS (63.8 % of those in the formoterol group, mean dose 452 μg [range 40–1600 μg] budesonide equivalents; 64.1 % in the salbutamol group, mean dose 477 μg [range 40–1600 μg] budesonide equivalents). LABAs were used by 5.4 % and 6.4 %, respectively, while oral β_2_-agonists/xanthines were used by 43.2 % and 44.3 % of patients, respectively (at the time this study was conducted, xanthines were considered to be an effective and inexpensive treatment for asthma, ideal for use in countries with more limited resources; hence, the high level of baseline use reported here).

The use of muscarinic receptor antagonists as concomitant medication was permitted during the study; however, there is no specific information on usage of this medication type prior to randomization.

### Safety

#### AEs

There was no significant difference in the percentage of patients reporting AEs in the formoterol or salbutamol groups (21.3 % vs 20.9 % of patients; OR 1.02, 95 % CI 0.85, 1.23; *p* = 0.81, Table 2). Total AEs included asthma-related AEs, non-asthma-related AEs and cardiovascular-related AEs.

**Table 2:** Adverse event (AE) data and analysis

AEs	Formoterol	Salbutamol	Odds ratio	*P* value
	(*n* = 1402)	(*n* = 1393)	(95 % CI)	
Total AEs	298 (21.3)	291 (20.9)	1.02 (0.85, 1.23)	0.813
Asthma-related AE	56 (4.0)	69 (5.0)	0.80 (0.56, 1.15)	0.221
Non-asthma-related AE	242 (17.3)	222 (15.9)	1.10 (0.90, 1.34)	0.347
Cardiovascular-related AE	7 (0.5)	12 (0.9)	0.58 (0.23, 1.47)	0.250
Non-cardiovascular-related AE	291 (20.8)	279 (20.0)	1.05 (0.87, 1.26)	0.633

Data are presented as n (%) patients

CI, *confidence interval*

#### SAEs and/or DAEs

There was no significant difference in the proportion of patients reporting SAEs and/or DAEs between the two groups (4.6 % of formoterol- vs 5.5 % of salbutamol-treated patients; OR 0.84, 95 % CI 0.60, 1.18; *p* = 0.323, Table 3). SAEs were reported by a similar proportion of patients in the formoterol and salbutamol groups (3.8 % vs 4.7 %, respectively), as were DAEs (1.1 % vs 1.2 %). There were no significant differences between groups for the proportion of asthma-related DAEs (0.6 % vs 0.3 %), non-asthma-related DAEs (0.4 % vs 0.9 %), SAE-related DAEs (0.2 % vs 0.4 %) or non-SAE-related DAEs (0.9 % vs 0.8 %).

**Table 3:** SAE/DAE data and analysis

Variable	Formoterol	Salbutamol	Odds ratio	*P* value
	(*n* = 1402)	(*n* = 1393)	(95 % CI)	
Total SAE and/or DAEs	65 (4.6)	76 (5.5)	0.84 (0.60, 1.18)	0.323
Serious adverse events	53 (3.8)	65 (4.7)	0.80 (0.55, 1.16)	0.245
Deaths	2 (0.1)	4 (0.3)	0.50 (0.09, 2.71)	0.419
Asthma-related SAE	41 (2.9)	51 (3.7)	0.79 (0.52, 1.20)	0.276
Non-asthma-related SAE	12 (0.9)	14 (1.0)	0.85 (0.39, 1.85)	0.682
Cardiovascular-related SAE	1 (0.1)	5 (0.4)	0.20 (0.02, 1.70)	0.140
Discontinuations due to AE	15 (1.1)	17 (1.2)	0.88 (0.44, 1.76)	0.709
Asthma-related DAE	9 (0.6)	4 (0.3)	2.24 (0.69, 7.30)	0.180
Non-asthma-related DAE	6 (0.4)	13 (0.9)	0.46 (0.17, 1.20)	0.113
SAE-related DAE	3 (0.2)	6 (0.4)	0.50 (0.12, 1.99)	0.322
Non-SAE-related DAE	12 (0.9)	11 (0.8)	1.09 (0.48, 2.47)	0.846

Data are presented as n (%) patients

CI, *confidence interval*

### Efficacy

#### Exacerbations

**Fig. 1:**
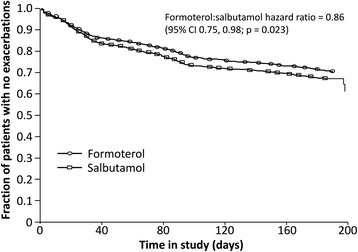
Kaplan–Meier plot of time to first exacerbation

**Fig. 2:**
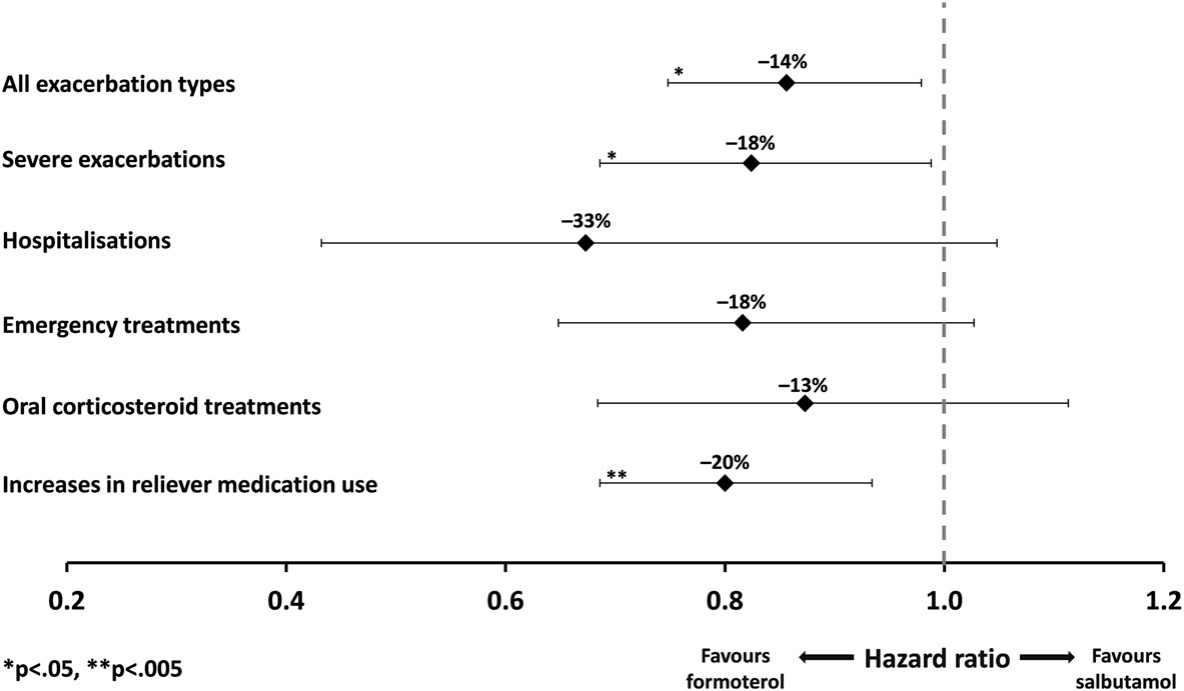
Reduction in exacerbation risk with formoterol versus salbutamol (%)

Compared with salbutamol, treatment with formoterol was associated with a 14 % longer time to first exacerbation (HR 0.86; 95 % CI 0.75, 0.98; *p* = 0.023, Fig. 1), which also corresponds to a 14 % reduction in overall exacerbation risk (*p* < 0.05, Fig. 2). The risk of severe exacerbations was 18 % lower with formoterol than salbutamol (OR 0.82, 95 % CI 0.69, 0.99; *p* = 0.037. Fig. 2). There was a significantly lower (−20 %) risk of requirement of increased asthma medication on formoterol compared with salbutamol (HR 0.80; 95 % CI 0.69, 0.93; *p* < 0.005).

There was a non-significant difference in the proportion of patients who experienced hospitalizations favouring formoterol (*n* = 33 [2.4 %] and 48 [3.4 %], respectively, *p* = 0.08 vs salbutamol). Emergency treatments (*n* = 133 [9.5 %] and 149 [11.4 %], *p* = 0.84) or oral corticosteroid treatments (*n* = 123 [8.8 %] and 138 [9.9 %], *p* = 0.27) were not different between treatments.

#### Use of formoterol or salbutamol

Over the course of the study, mean daily reliever medication use decreased in both treatment groups (Table 4) but was significantly lower with formoterol compared with salbutamol during all three study periods. The difference was −0.20 (95 % CI −0.37, −0.04; *p* = 0.017) at the end of Period 1 and −0.31 (95 % CI −0.48, −0.14; *p* < 0.001) at the end of Period 2; the mean difference then remained relatively stable until the end of Period 3 (−0.30; 95 % CI −0.47, −0.13; *p* < 0.001, Table 4). Compared with salbutamol, formoterol was associated with 20 % less study medication use. While patients in the formoterol group had numerically fewer asthma symptom days than patients using salbutamol, the difference between groups was not significant (*p* ≥ 0.24, Table 4).

**Table 4:** Analysis of study medication use and days with asthma symptoms

	Formoterol	Salbutamol	Mean difference	*P* value
	(*n* = 1402)	(*n* = 1393)	(95 % CI)	
Use of study medication, doses/day (adjusted mean)				
Period 1	1.62	1.83	−0.20 (−0.37,−0.04)	0.017
Period 2	1.52	1.83	−0.31 (−0.48,−0.14)	< 0.001
Period 3	1.36	1.66	−0.30 (−0.47,−0.13)	< 0.001
Asthma symptom days, % (adjusted mean)				
Period 1	43.5	44.4	−0.92 (−3.92, 2.08)	0.55
Period 2	43.1	44.5	−1.41 (−4.42, 1.61)	0.36
Period 3	39.1	40.9	−1.83 (−4.86, 1.19)	0.24

CI, *confidence interval*

## Discussion

The real-world global RELIEF study compared formoterol and salbutamol as reliever medication in a diverse group of patients with varying degrees of asthma severity and using a variety of maintenance treatments [5]. The RELIEF study found that formoterol was at least as safe as salbutamol when used as reliever medication, and that finding was reproduced during this *post-hoc* analysis of the East Asian patient subgroup. While the overall findings for both analyses are similar, key differences were noted between the full patient population and this subgroup. In the full study, significantly fewer asthma-related AEs and non-asthma-related AEs such as headaches, tremor and allergic rhinitis were reported for formoterol compared with salbutamol; however, there were also a significantly greater number of total DAEs (caused by non-serious AEs), asthma-related DAEs and non-asthma-related DAEs (including tremor, headache and tachycardia) reported for formoterol compared with salbutamol. By contrast, the East Asian subgroup analysis found no significant differences in risk of any AE (including SAEs and DAEs) between formoterol and salbutamol reliever treatments. There was a significant reduction in the risk of any exacerbation (14 %) and severe exacerbations (18 %) with use of formoterol compared with salbutamol. Formoterol treatment significantly increased the time to first exacerbation and reduced the need for additional reliever medication compared with salbutamol (−20 %).

The primary results of the global RELIEF study showed that the proportion of patients with an AE was similar between treatment groups (42 % of patients in both formoterol and salbutamol groups experienced at least one AE) as was the proportion of patients experiencing SAEs (3.1 % and 3.3 %, respectively). The most commonly reported AEs included aggravated asthma (12–13 % of each patient group) and nasopharyngitis (5 %). The time to first exacerbation was longer with formoterol (HR 0.86) and 16 % less as-needed medication was used in the formoterol group in the final treatment period [5]. In this subgroup of patients in East Asia, the results for SAEs (3.8 % and 4.7 %, respectively), time to first exacerbation (HR 0.86) and as-needed medication use (reliever use for formoterol was 18 % lower than salbutamol in the final treatment period) all favoured formoterol to a similar, if not the same, extent as in the main study population. Interestingly, only 21 % of patients recruited from East-Asian countries reported an AE compared with 42 % of the global RELIEF study population. It is possible that this result reflects cultural differences in study populations with regard to reporting events.

It should also be noted that the East Asian patient subgroup had high baseline levels of xanthine/oral β_2_-agonist use (almost half of all patients); this information, in conjunction with the low AE rate reported in this subanalysis, suggests the relative safety of the addition of formoterol to concomitant xanthine/oral β_2_-agonist and maintenance therapy. This cannot be confirmed, however, as the analysis was not designed to investigate this point, and the potential influence of cultural differences (as stated above) should also be considered.

The results of the global RELIEF study and this subgroup analysis of patients in East Asia are consistent with those described in the literature, in which more favourable efficacy outcomes with formoterol and similar safety profiles have been reported (compared with salbutamol) and no significant differences observed in subgroups from Asia [4, 6, 7]. A Cochrane review of formoterol treatment in chronic asthma found that compared with regular salbutamol or terbutaline, there was no significant increase in fatal or non-fatal SAEs with formoterol [6]. Another Cochrane review compared formoterol and SABAs when used as reliever medication in adults and children with asthma [4]. This review grouped all SABAs and found a significant effect in favour of formoterol versus any SABA for an exacerbation that was treated with oral corticosteroids. While there were fewer SAEs in patients who received formoterol, this did not reach statistical significance versus SABAs [4].

Recent data have shown that corticosteroid insensitivity may be reversed by formoterol treatment. The actions of formoterol and salbutamol were compared in an *in vitro* study using peripheral blood mononuclear cells from patients with severe asthma or COPD [8]. Formoterol was found to reverse corticosteroid insensitivity in both cell types, while salbutamol was found to be effective only in cells from patients with severe asthma. Although these data are preliminary, this may be of interest when considering the suitability of formoterol treatment for patients with severe asthma or asthma-COPD overlap syndrome.

The uniformity of results between the global RELIEF population and the East Asia subgroup is consistent with a sub-analysis of the COSMOS study, where the efficacy and safety of budesonide/formoterol maintenance and reliever therapy was compared with salmeterol/fluticasone propionate plus as-needed salbutamol [7]. Outcomes observed in patients enrolled across East-Asian countries (specifically China, Korea, Taiwan and Thailand) were consistent with those in the overall study population [5].

Strengths of the RELIEF study included minimal entry criteria (with respect to age, comorbidities and concomitant medications) as well as the absence of a run-in period and lung function or compliance monitoring. These features were deliberately chosen to maximise patient recruitment and ensure that the patient population and procedures reflected everyday clinical practice. This study also comprised a large patient population and was of a sufficient duration to detect treatment effects. The open-label nature of the study means that the data can be generalised to the real-world population of patients being treated in everyday clinical practice.

Limitations of the study design include the fact that the primary inclusion criterion for this subgroup analysis was based on residence and patients were not recruited based on ethnic origin. However, a very small proportion of patients in this analysis were not of Oriental descent, and it is unlikely that the results were skewed by the inclusion of 48 patients who were of Caucasian or other descent. As with any *post-hoc* analysis, the analysis is limited to the data that were originally collected. Such data could contain factors that are not apparent and could influence the results (e.g. patient characteristics or genetic factors that were not assessed).

The study investigators were permitted to adjust maintenance treatment as required according to clinical need, and any increase in asthma medication was defined as an exacerbation. Given the open-label nature of the study design there was consequently the potential for bias, as investigators had the option to increase asthma medication and consequently increase the apparent exacerbation rate.

The lack of information on patient adherence to treatment is another limitation of this study. Since treatment was provided as required, it would be difficult to distinguish between non-adherent patients (e.g. patients experiencing symptoms, but not using their medication or with few symptoms but over-using their medication) and adherent patients (e.g. those with fewer symptoms who have a reduced need for as-needed treatment or *vice versa*). Therefore, adherence monitoring was not attempted. However, as stated previously, the advantages of an open-label study (wide applicability to real-world practice) were thought to outweigh these potential limitations.

It is now widely acknowledged that when used alone, LABAs are associated with risks of increased exacerbations and increased mortality in patients with asthma compared with ICS/LABA [9, 10]. However, the addition of a LABA to an ICS is beneficial and the risk of death, intubation or hospital admissions for exacerbations is not increased with combination LABA and ICS therapy versus ICS alone [11]. All guidelines stress that LABAs should not be used without concomitant ICS treatment [9–11]. In the time since the main RELIEF study was conducted, the treatment landscape has changed and LABA-only regimens are not recommended in patients with asthma. Hence further studies using only formoterol or salbutamol will not be conducted. However, similar *post-hoc* analyses may help to elucidate whether or not differences exist between other regional or ethnic subgroups and broad, mixed populations.

## Conclusion

This *post-hoc* analysis of the global RELIEF study demonstrates that among patients with asthma in East Asia, as-needed formoterol and salbutamol had similar safety profiles but formoterol reduced the risk of exacerbations, increased the time to first exacerbation and reduced the need for reliever medication compared with salbutamol.

## Additional file

Additional file 1:
**Supplementary Tables 1 and 2.** (DOCX 19 kb)

## Abbreviation

AE: Adverse event
CI: Confidence interval
DAE: Discontinuation due to adverse event
DPI: Dry powder inhaler
GINA: Global INitiative for Asthma
HR: Hazard ratio
ICS: Inhaled corticosteroid
LABA: Long-acting β_2_-agonist
OR: Odds ratio
RELIEF: REal Life EFfectiveness of Oxis® Turbuhaler® as-needed in asthmatic patients
SABA: Short-acting β_2_-agonist
SAE: Serious adverse event

## References

[1] Global Initiative for Asthma. Global Strategy for Asthma Management and Prevention. 2015. http://www.ginasthma.org/local/uploads/files/GINA_Report_2015_Aug11. (Accessed 5th January 2016).

[2] Tattersfield AE, Lofdahl CG, Postma DS, Eivindson A, Schreurs AG, Rasidakis A (2001). Comparison of formoterol and terbutaline for as-needed treatment of asthma: a randomised trial. Lancet.

[3] Ind PW, Villasante C, Shiner RJ, Pietinalho A, Boszormenyi NG, Soliman S (2002). Safety of formoterol by Turbuhaler as reliever medication compared with terbutaline in moderate asthma. Eur Respir J.

[4] Welsh EJ, Cates CJ. Formoterol versus short-acting beta-agonists as relief medication for adults and children with asthma. The Cochrane database of systematic reviews. 2010;(9):CD008418. doi:10.1002/14651858.CD008418.pub2 10.1002/14651858.CD008418.pub2 PMC4034434 20824877

[5] Pauwels RA, Sears MR, Campbell M, Villasante C, Huang S, Lindh A (2003). Formoterol as relief medication in asthma: a worldwide safety and effectiveness trial. Eur Respir J.

[6] Cates CJ, Cates MJ (2012). Regular treatment with formoterol for chronic asthma: serious adverse events. Cochrane Database Syst Rev.

[7] Vogelmeier C, Naya I, Ekelund J (2012). Budesonide/formoterol maintenance and reliever therapy in Asian patients (aged >/=16 years) with asthma: a sub-analysis of the COSMOS study. Clin Drug Investig.

[8] Rossios C, To Y, Osoata G, Ito M, Barnes PJ, Ito K (2012). Corticosteroid insensitivity is reversed by formoterol via phosphoinositide-3-kinase inhibition. Br J Pharmacol.

[9] Chowdhury BA, Dal PG (2010). The FDA and safe use of long-acting beta-agonists in the treatment of asthma. N Engl J Med.

[10] Cockcroft DW, Sears MR (2013). Are inhaled longacting beta2 agonists detrimental to asthma?. Lancet Respir Med.

[11] Levenson M. Long-acting beta-agonists and adverse asthma events meta-analysis. Statistical Briefing Package for Joint Meeting of the Pulmonary-Allergy Drugs Advisory Committee, Drug Safety and Risk Management Advisory Committee and Pediatric Advisory Committee on December 10–11, 2008

