# Supplementary material for: Formoterol as reliever medication in asthma: a post-hoc analysis of the subgroup of the RELIEF study in East Asia
Source: BMC Pulm Med. 2016 Jan 12;16:8. doi: 10.1186/s12890-015-0166-0 (PMC4711052; doi:10.1186/s12890-015-0166-0)
Supplement: Additional file 1: — Supplementary Tables 1 and 2. (DOCX 19 kb) [file 12890_2015_166_MOESM1_ESM.docx]

**Additional material**

**Supplementary Table 1. Details of study centres included in this analysis**

| **Primary facility** | **Primary investigator(s)** |
| --- | --- |
| 304 Hospital | Y Guo |
| Anzhen Hospital, Capital University of Medical Science | Y Liang |
| Beijing Children’s Hospital | S Liu |
| Beijing Ji Shui Tan Hospital | D Wang |
| Bj Children Research Institute | Y Chen |
| Chang Hai Hospital/2^nd^ Military Medical University | Q Li |
| Chang Zeng Hospital/2^nd^ Military Medical University | Q Xiu |
| Children’s Hospital | J Zhao |
| Children’s Hospital/Su Zhou Medical University | J Sheng |
| Gu Lou Hospital | L Dai |
| H Z No. 1 People’s Hospital | J Zhu |
| No. 1 People’s Hospital | F Hui |
| No. 3 Hospital of Beijing Medical University | M Zhao, M Li |
| No. 6 People’s Hospital | A Shen |
| No. 9 People’s Hospital | H Gu |
| People’s Hospital of Jiangsu Province | M Huang |
| PLA General Hospital of Navy | H Wang |
| Pulmonary Disease Hospital | H Li |
| Ren Ji Hospital | Y Xu |
| S M U Affiliated Pediatric Hospital | L Zhang |
| Shanghai Children’s Hospital | Q Lu |
| Shanghai Construction Hospital | X Wang |
| Shanghai First People’s Hospital | X Zhou, J Hong |
| Shanghai Hua Shan Hospital/Shanghai Medical University | X Chen |
| Shanghai Rui Jin Hospital/Shanghai 2nd Medical University | S Huang, Y Li |
| Shanghai Texitile No.1 Hospital | H Zhang |
| Shanghai Zhong Shen Hospital | S Niu |
| Sino-Japanese Friendship Hospital | J Lin |
| T J M U No. 2 Affiliated Hospital | C Liu |
| Tianjin Chest Hospital | L Pang |
| Tianjin General Hospital | B Chen |
| Xin Hua Hospital/Shanghai Second Medical University | J Lin |
| Z J M U No. 1 Affiliated Hospital | J Zhou |

**Supplementary Table 2. Demographics of other regional subgroups and of the entire study population (Pauwels et al 2003)**

|  | **Western region**  **(n=12,265)** | **Eastern region**  **(n=2834)** | **Other region**  **(n=3025)** | **Full cohort [**[**5**](#_ENREF_5)**]**  **(N=18,124)** |
| --- | --- | --- | --- | --- |
| Male | 5079 (41.4) | 1436 (50.7) | 1225 (40.5) | 7793 (43.0) |
| Mean age, years (range) | 41 (4–91) | 35 (5–85) | 32 (5–82) | 39 (4–91) |
| Age groups | | | | |
| Children  (≤11 years) | 590 (4.8) | 512 (18.1) | 594 (19.6) | 1696 (9.4) |
| Adolescents  (12–17 years) | 961 (7.8) | 232 (8.2) | 401 (13.3) | 1594 (8.8) |
| Adults  (18–64 years) | 9200 (75.0) | 1903 (67.1) | 1891 (62.5) | 12994 (71.7) |
| Elderly  (≥65 years) | 1514 (12.3) | 187 (6.6) | 139 (4.6) | 1840 (10.2) |
| Race | | | | |
| Black | 72 (0.6) | 0 (0) | 96 (3.2) | 168 (0.9) |
| Caucasian | 12061 (98.3) | 12 (0.4) | 1774 (58.6) | 13817 (76.2) |
| Oriental | 74 (0.6) | 2786 (98.3) | 6 (0.2) | 2866 (15.8) |
| Other | 58 (0.5) | 36 (1.3) | 1179 (39.0) | 1273 (7.0) |
| Asthma severity (judged by asthma medication level)* | | | | |
| Intermittent | 1627 (13.3) | 407 (14.4) | 789 (26.1) | 2823 (15.6) |
| Mild | 3781 (30.8) | 1453 (51.3) | 1079 (35.7) | 6313 (34.8) |
| Moderate | 4776 (38.9) | 617 (21.8) | 888 (29.4) | 6281 (34.7) |
| Severe | 2081 (17.0) | 357 (12.6) | 269 (8.9) | 2707 (14.9) |

Data are presented as n (%) patients, unless otherwise stated.

* Intermittent: no maintenance treatment; mild: inhaled corticosteroid (ICS) <500 μg/day (<400 μg/day in children) or a regular long-acting β_2_-agonist (LABA), cromone, theophylline or leukotriene modifier; moderate: ICS alone at any dose ≥500 μg/day (≥400 μg/day in children), or ICS 500–800 μg/day (400–800 μg/day in children) in combination with LABA, theophylline or leukotriene modifier; severe: ICS >800 μg/day in combination with LABA, theophylline, leukotriene modifier, or oral corticosteroids.

Pauwels RA, Sears MR, Campbell M, Villasante C, Huang S, Lindh A et al. Formoterol as relief medication in asthma: a worldwide safety and effectiveness trial. The European respiratory journal. 2003;22(5):787-94.
